# A Rapid Systematic Review Exploring the Involvement of Medical Students in Pandemics and Other Global Health Emergencies

**DOI:** 10.1017/dmp.2020.315

**Published:** 2020-09-02

**Authors:** Anastasia Martin, Iris Martine Blom, Gemma Whyatt, Raghav Shaunak, Maria Inês Francisco Viva, Lopamudra Banerjee

**Affiliations:** 1 King’s College London, London, United Kingdom; 2 University of Amsterdam, Amsterdam, The Netherlands; 3 University of Leeds, Leeds, United Kingdom; 4 Universidade Nova de Lisboa, Lisbon, Portugal; 5 Cardiff University, Cardiff, United Kingdom

**Keywords:** COVID-19, health emergencies, medical student, pandemic, rapid systematic review role

## Abstract

**Objectives:**

The role of medical students in the current coronavirus disease 2019 (COVID-19) pandemic is rapidly evolving. The aim of this review is to explore the involvement of medical students in past global health emergencies, to help inform current and future scenarios.

**Methods:**

A rapid systematic review was undertaken, including articles from online databases discussing the roles, willingness and appropriateness of medical student involvement in global health emergencies. Data were extracted, appraised and written up as a narrative synthesis. This study was registered with PROSPERO (CRD42020177231).

**Results:**

Twenty-eight articles were included. Medical students played a wide variety of clinical and nonclinical roles including education and logistics, although medical assistance was the most commonly reported role. Challenges included a lack of preparedness and negative mental health impacts. A total of 91.7% of included articles about willingness found medical students were more willing to be involved than not.

**Conclusions:**

This review shows medical students are capable and willing to be involved in global health emergencies. However, there should be clear protocols for the roles that they play, taking into account the appropriateness. As a rapid review, there were study limitations and more research is required regarding the impact of these roles on medical students and the system.

Medical students represent a largely untapped reservoir of potential in global health issues. This group can provide a youth perspective to global health issues, contribute to research in a manner in which busy seniors cannot, and be an addition to the global health workforce in times of need.^1,2^ This last point is the focus of our research.

At the time of writing (April 2020), the world was experiencing coronavirus disease 2019 (COVID-19); a pandemic with unprecedented social and financial impacts that was placing pressure on healthcare systems around the world. One solution to alleviating this pressure was by asking medical students to help.^3-5^

Many medical schools have been suspended or moved to online platforms, leaving students with extra time on their hands; time that can potentially be spent helping.^4,5^ Medical students worldwide have already started moving to the frontline.^6^ In the United Kingdom and Brazil, for example, final year medical students have been fast-tracked to join the workforce early.^7,8^

Guidance toward medical students helping on the frontline has been published by several organizing bodies, but due to the dynamic situation, this guidance continues to change.^9,10^ Therefore, it is important to ascertain what exactly would be the best way for medical students to help. The primary role of the medical student is to learn to be a doctor, and deviations from this may have consequences both for medical students and the healthcare system around them. However, having students in the healthcare environment during a pandemic can be an increased burden to clinicians who need to invest time to teach them. Consequently, students’ education and patients’ care may be compromised.^11^

Currently, there are multiple articles describing the role of medical students in past similar scenarios, their willingness to assist, as well as opinions on what roles medical students should play during COVID-19.^12,13^ However, to our knowledge, a systematic review that combines and assesses this information does not currently exist. Filling this gap in the evidence base will help better inform what role medical students can play in future global health emergencies, including COVID-19.

The aim of this review was to systematically assess how medical students can be involved in pandemics and global health emergencies. By outlining the roles medical students have already undertaken or potentially can undertake, the appropriateness of these roles, as well as how willing medical students are to be involved, this review can be used to guide decision-makers to design safe and effective roles for medical students in current and future global health emergencies.

## METHODS

A rapid systematic review was used to collate, critically appraise, and synthesize the information, because it answered our question in a timely manner so our research could potentially inform the COVID-19 pandemic. PRISMA guidelines were followed throughout the process.^14^ The review protocol submitted on March 30, 2020, accepted April 20, 2020 and can be found in the PROSPERO database [number: CRD42020177231].

### Search Strategy

Four independent reviewers (A.M., R.S., I.B., I.V.) conducted a systematic search on March 31, 2020. PubMed, MEDLINE, Embase, and the Global health database were searched for eligible studies with no date restrictions. Studies were available from 1946 to March 2020. The full search strategy can be found in Appendix 1. Bibliographies from eligible articles were screened (ie, snowballing) and the “Journal of Disaster Medicine and Public Health Preparedness” was hand searched through all volumes for further articles by reviewer L.B. Papers were initially screened by title and abstract and shortlisted articles were screened for full-text analysis against eligibility criteria by all 6 reviewers and any disputes were discussed. Data from eligible articles were inputted into a tailored data collection form which was trialed by 2 reviewers before use. All steps were recorded using the PRISMA diagram.

### Eligibility Criteria

All studies that discussed roles medical students have played or can play, their willingness to do so, or whether they should do so in an acute global health emergency, including hypothetical studies, were included. “Global health emergency” was defined as any event that significantly and acutely affected the capacity or functioning of a health system in 1 or more countries, including but not limited to: infectious disease outbreaks, natural or man-made disasters, and armed conflict.

Only English language articles were included. The population of interest was medical students, defined as anyone from any country enrolled in a university course (undergraduate or postgraduate) training to be a doctor who has not yet finished their medical education. Articles that involved other participants (eg, nursing students) but also mentioned medical students, were included.

Qualitative research, quantitative research, and mixed methods studies, including systematic, scoping and literature reviews, published editorials, commentaries, and conference abstracts were included. We included gray literature to add a wider perspective. Gray literature included unpublished or nonpeer reviewed papers, reports, theses, and technical documentation.^15^

Studies involving nonacute events and diseases that were deemed nongeneralizable to the COVID-19 outbreak, eg, the opioid epidemic and obesity crisis, were excluded. Furthermore, studies that did not discuss medical students, eg, studies that exclusively discussed other populations (eg, veterinary, dental, or nursing students) were excluded because they did not include our population of interest. Non-English language papers were also excluded due to time constraints of translating these papers.

### Outcome Measures

Our primary outcome was to collate descriptions of the roles medical students can play in a pandemic. These were predefined in the data extraction proforma as: clinical assistance; testing; helplines; triage; raising awareness; or “other,” if a different role was described, which the reviewer then specified. Our secondary outcomes included the level of willingness of medical students to help, factors affecting willingness, and appropriateness of the role.

### Data Extraction and Analysis

Data were extracted using the predefined standardized form and included: article, author, year, journal, country, article type, article design, aim, area studied (role/willingness/appropriateness/preparedness), article population, setting/context, the number of participants, methodology, outcomes, key findings, relation to past global health emergency (if applicable), comments on the role; comments on willingness, comments on appropriateness, and critical appraisal.

Articles were critically appraised globally and briefly judged for risk of bias; however, the full use of quality assessment tools was not feasible due to time constraints. All included papers were critically appraised using the reviewers’ judgment, expertise, and by means of discussion among the 6 researchers. The design, outcome measure, and whether the study was peer-reviewed or a gray literature study played a role in guiding whether articles are of high or low quality and are reported on in the discussion section.

Data were analyzed thematically and written up as a narrative synthesis.

## RESULTS

### Article Characteristics

**FIGURE 1:**
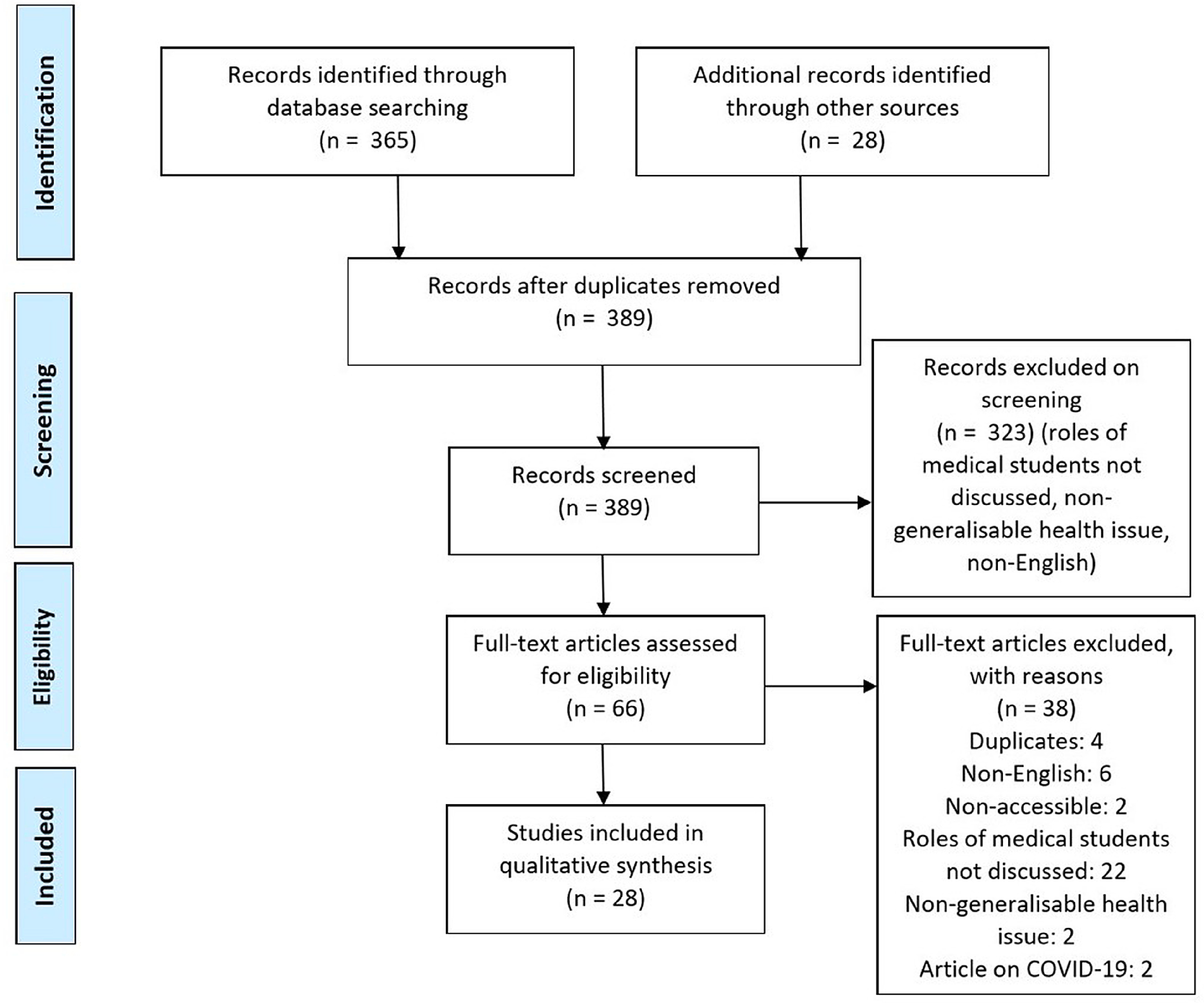
PRISMA flow diagram of articles identified, screened, included and excluded.

Our search identified 802 articles as well as 28 from extra sources. A total of 365 articles were screened for title and abstract, and 323 of those were excluded. Sixty-six articles were screened for the full manuscript, and 28 of those met the inclusion criteria (Figure 1). Most of the included articles had good quality of evidence but unpublished gray literature (eg, expert opinion pieces) had poorer evidence quality.

Table 1 shows the baseline characteristics of all included articles. Seventeen articles discussed roles, 12 discussed willingness, and 13 directly discussed appropriateness (Chen E, Goodman KW, Fiore RN. Involving medical students in disaster response: ethics, education and opportunity [unpublished PDF]. 2014;1-17).^16-42^ As well as 13 articles purely discussing medical students, articles also examined nursing, pharmacy, and dental students. The views of medical students across multiple years were examined with an even spread. The context of the global health emergencies is detailed in Table 1, with 13 articles reporting on past situations that happened and 15 articles discussing hypothetical scenarios (Table 1).

**TABLE 1:** Baseline Characteristics of All Included Articles

Article ID	Location of publication	Article Type	Main Theme(s)	Hypothetical or Real Emergency	Global Health Emergency Context
Abud (2015)^16^	USA	Abstract	Role	Real	Ebola outbreak
Al-Ziftaw (2020)i^17^	Qatar	Cross-sectional survey	Willingness, preparedness	Hypothetical	General disaster events
Ayaz Sabri (2006)^18^	Pakistan	Commentary	Role	Real	Earthquake
Berhane (2010)^19^	USA	Cross-sectional survey	Role, willingness	Hypothetical	H1N1 Influenza pandemic
Boulos (2014)^20^	USA	Abstract	Role	Hypothetical	Anthrax
Chen (Unpublished PDF)	USA	Essay	Role	Mixed	Mixed
Cohen (1991)^21^	USA	Commentary	Role	Real	HIV epidemic
Eastwood (2006)^22^	USA	Commentary	Role, appropriateness	Mixed	SARS epidemic
Gouda (2019)^23^	Ireland	Cross-sectional survey	Willingness	Hypothetical	Mixed disasters (natural disaster and infectious epidemic)
Herman (2007)^24^	Canada	Cross-sectional survey	Willingness, appropriateness, preparedness	Hypothetical	Influenza Pandemic
Hwang (2014)^25^	Korea	Cross-sectional survey	Willingness, appropriateness	Hypothetical	Infectious disease outbreak
Kaiser (2009)^26^	USA	Cross-sectional survey	Willingness, preparedness	Hypothetical	Mixed disasters regardless of severity (natural disaster and influenza pandemic)
Kaiser (2011)^27^	USA	Commentary	Role, appropriateness	Hypothetical	H1N1 outbreak
Katz (2002)^28^	USA	Cross-sectional survey	Role	Real	Terrorist attack
Keil (2007)^29^	UK	Commentary	Role, appropriateness, preparedness	Mixed	H5N1 pandemic
Lim (2009)^30^	Singapore	Commentary	Role, appropriateness, preparedness	Real	Viral pandemic
Lin (2009)^31^	USA	Cross-sectional survey	Role	Hypothetical	Not specified
Markenson (2013)^32^	USA	Cross-sectional survey	Willingness, preparedness	Hypothetical	General disaster events
Mortelmans (2009)^33^	Belgium	Cross-sectional survey	Willingness	Hypothetical	H5N1 pandemic
Mortelmans (2013)^34^	Belgium	Cross-sectional survey	Willingness, preparedness	Hypothetical	Mixed (nuclear, chemical, biological, very infectious outbreak (eg, H5N1), very dangerous contagious outbreak (eg, Ebola)
Mortelmans (2015)^35^	The Netherlands	Cross-sectional survey	Willingness	Hypothetical	Infectious disease outbreak of varying severity
Patel (2017)^36^	USA	Cross-sectional survey	Willingness	Hypothetical	Infectious disease outbreak
Patil (2003)^37^	Hong Kong	Commentary	Role	Real	SARS epidemic
Rega (2011)^38^	USA	Commentary	Role, appropriateness, preparedness	Real	H1N1 outbreak
Reyes (2010)^39^	Chile	Commentary	Role, appropriateness, preparedness	Real	Earthquake and tsunami
Starr (1976)^40^	USA	Essay	Role, preparedness	Real	Spanish flu
Tebruegge (2010)^41^	Australia	Cross-sectional survey	Willingness	Hypothetical	H1N1
Trepman (2001)^42^	Canada	Essay	Role	Real	WWII concentration camp

### Role

Seventeen of the 28 included articles discussed the roles of medical students during global health emergencies (Table 2). The most commonly reported role was providing clinical assistance (*n* = 12). In 2 of these articles, medical students acted as junior doctors.^18,40^ In 1918, during the outbreak of the Spanish Flu, medical students acted as nurses and interns because the usual medical workforce were away assisting the war troops.^40^ During the Kashmir Earthquake in Pakistan, medical students were sent to less accessible places compared with senior doctors due to their young age.^18^

**TABLE 2:** Overview of the Described Roles of Medical Students in Global Health Emergencies

Article ID	Method of Reporting Role	Number of Participants	Roles Reported	Difference in Years of Medical Study	Hypothetical or Real Emergency	Global Health Emergency Context
Abud (2015)^16^	Report of events	N/A	Raising awareness on social media	N/A	Real	Ebola outbreak
Ayaz Sabri (2006)^18^	Report of events	N/A	Acting as doctors	The article only describes final year students acting as doctors	Real	Earthquake
Berhane (2010)^19^	Questionnaire	375	Patient care, volunteer	Pre-clinical year students reported a lack of clinical skills more often as a reason not to participate in patient care	Hypothetical	H1N1 influenza pandemic
Boulos (2014)^20^	Simulation	N/A	Distributing medication	N/A	Hypothetical	Anthrax
Chen (Unpublished PDF)	Perspective	N/A	Triage, assist with life-saving procedures	N/A	Mixed	Mixed
Cohen (1991)^21^	Report of events	N/A	Raising awareness	N/A	Real	HIV epidemic
Eastwood (2006)^22^	Perspectives	N/A	Clinical assistance	In 1 medical school, it was decided that only final-year medical students should be able to provide care to patients with SARS	Mixed	SARS epidemic
Kaiser (2011)^27^	Perspective	N/A	Clinical assistance, helplines, triage	First-year students for helplines and vaccination; more clinically proficient students should perform more clinically relevant duties	Hypothetical	H1N1 outbreak
Katz (2002)^28^	Questionnaire	157	Crisis hotline, fundraising, volunteering with psychiatric disaster outreach, clinical assistance, blood donation, food preparation for rescue workers	Students further in their medical education were more likely to provide clinical assistance	Real	Terrorist attack
Keil (2007)^29^	Perspective	N/A	Clinical assistance, porters, giving information, observations, telephone, triage, clerking, staff immunization	N/A	Mixed	H5N1 pandemic
Lim (2009)^30^	Literature review	N/A	Education	N/A	Mixed	Viral pandemic
Lin (2009)^31^	Questionnaire	31	Manual ventilation, medical assistance, cardiopulmonary resuscitation	N/A	Hypothetical	N/A
Patil (2003)^37^	Report of events	Over 200 medical and nursing students	Raising awareness	N/A	Real	SARS epidemic
Rega (2011)^38^	Perspective	10	Registration, documentation, traffic control,patient surveillance, and education, immunizations	N/A	Real	H1N1 outbreak
Reyes (2010)^39^	Perspective	N/A	Clinical assistance, triage	N/A	Real	Earthquake and tsunami
Starr (1976)^40^	Perspective	N/A	Acting as medical interns and nurses	Fourth-year medical students acted as interns and third-year medical students acted as nurses	Real	Spanish flu
Trepman (2001)^42^	Literature review	95-100	Clinical assistance, logistics set-up emergency hospital, social-relief work	N/A	Real	WWII concentration camp

Other noteworthy cases include how, in the 1950s, during the poliomyelitis outbreak in Europe, 1500 medical and dental students provided mechanical ventilation (Chen E, Goodman KW, Fiore RN. Involving medical students in disaster response: ethics, education and opportunity [unpublished PDF]. 2014;1-17). In 2001, after the World Trade Center (WTC) terrorist attack, students provided clinical assistance in hospital emergency wards and participated in surgical and resuscitation teams.^28^ In 2010, after an earthquake, a tsunami hit Chile and medical students provided medical care in remote areas.^39^ Recently, it has also been hypothesized that medical students can provide clinical care by distributing medication to hospital staff and providing manual ventilation.^20,31^

The role of medical students as educators through raising awareness was also highlighted (*n* = 3). In 1987, during the HIV epidemic, medical students developed a peer-to-peer teaching system to raise awareness and contribute to better patient care.^21^ During the severe acute respiratory syndrome (SARS) and Ebola outbreaks, students held (online) campaigns to promote healthier behavior.^16,37^

Other reported roles entailed nonclinical assistance. In 1945, in the immediate liberation of Belsen Concentration Camp, medical students set up an acute care hospital for emergency treatment of the inmates waiting to be transferred and a pharmaceutical dispensary.^42^ During the 2001 WTC terrorist attack, medical students participated as “runners” to carry information between triage stations, prepared food for rescue workers, worked in emergency hotlines to provide information to families of victims, fundraised, donated blood, and assisted in psychiatric disaster services (Chen E, Goodman KW, Fiore RN. Involving medical students in disaster response: ethics, education and opportunity [unpublished PDF]. 2014;1-17).^28^

Medical students who were further in their medical education were more likely to provide medical assistance (*n* = 6).^18,19,22,27,28,40^ Furthermore, the challenges faced by medical students in their roles during the global health emergency was discussed (*n* = 2). These challenges included: a lack of supervision, treating children, and prioritizing medical need.^18,40^

The impact on the mental health of medical students was also highlighted, both during and after the emergency event (*n* = 3).^28,29,39^ For example, during the provision of clinical care during the 1918 influenza epidemic, medical students experienced psychological distress during their fieldwork, such as fear, anxiety, depressive symptoms, despair, and panic.^40^ An article on the 2001 WTC terrorist attack suggested that the type of role played appeared to correlate with how much the students were affected. It was found that students working in fundraisers and hotlines had significantly higher symptoms. Medical students who assisted in medical care at hospitals had the fewest symptoms and a greater sense of empowerment.^28^ The same article also concluded that involvement in the disaster was associated with a reinforced desire to become a physician.^28^

### Willingness

Twelve of the included articles evaluated the willingness of medical students to help in disaster situations (Table 3). Most (*n* = 11) reported quantitative data on the level of willingness of medical students to help in a disaster as shown in Table 3. The percentage of medical students willing to help ranged between 36% and 92.5%, with 9 of the 11 articles reporting a willingness percentage greater than or equal to 50%. One article reported the willingness of medical students to be involved on a different scale but equated it as “moderate willingness.”^17^ One article did not provide quantitative data but reported that “the majority of students responding to the survey were willing to respond to disaster events.”^32^

**TABLE 3:** Overview of the Reported Willingness of Medical Students to Be Involved in the Response to Global Health Emergencies

Article ID	Method of Measuring Willingness	Number of Participants	Willingness Reported (% of Students Willing/ Points Scored on Willingness Survey)	Hypothetical or Real Emergency	Global Health Emergency Context
Al-Ziftawi (2020)^17^	Questionnaire	187	33.7 ± 8.99 points (out of 55) which equated to “moderate willingness to be involved”	Hypothetical	General disaster events
Berhane (2010)^19^	Questionnaire	375	86%	Real	H1N1 influenza pandemic
Gouda (2019)^23^	Questionnaire	274	Natural disaster: 69% Infectious epidemic: 59.1%	Hypothetical	Mixed disasters (natural disaster and infectious epidemic)
Herman (2007)^24^	Questionnaire	354	69.77%	Hypothetical	Influenza pandemic
Hwang (2014)^25^	Discussion based on novel and book reports	50	36%	Hypothetical	Infectious disease outbreak
Kaiser (2009)^26^	Questionnaire	523	Natural disaster: 92.5% Influenza pandemic: 87.8%	Hypothetical	Mixed disasters regardless of severity (natural disaster and influenza pandemic)
Markenson (2013)^32^	Questionnaire	136	“The majority of students responding to the survey were willing to respond to disaster events”	Hypothetical	General disaster events
Mortelmans (2009)^33^	Questionnaire	243	82.30%	Hypothetical	H5N1 pandemic
Mortelmans (2013)^34^	Questionnaire	1103	Very infectious: 77.6% Very dangerous and contagious: 70.4%	Hypothetical	Mixed (nuclear, chemical, biological, very infectious outbreak (e.g. H5N1), very dangerous contagious outbreak (eg, Ebola)
Mortelmans (2015)^35^	Questionnaire	999	Very Infectious: 75.4% Very dangerous and contagious 43.1%	Hypothetical	Infectious disease outbreak of varying severity
Patel (2017)^36^	Questionnaire	238	Respiratory transmission: 50% Contact transmission: 61%	Hypothetical	Infectious disease outbreak of varying transmission (if PPE were available)
Tebruegge (2010)^41^	Questionnaire	76	97.40%	Hypothetical	H1N1

Seven of the included articles provided reasons to explain the medical students’ level of willingness. Obligation or social responsibility was stated most commonly, along with altruism, as a reason why medical students were willing to be involved. Concerns for personal health and safety, as well as concerns for family health and safety, were the 2 most commonly stated reasons for students being unwilling to help (Table 4). Some (*n* = 8) also discussed factors that affected the willingness, which are described in Table 5.

**TABLE 4:** Summary of the Reasons Provided to Explain the Level of Willingness Reported by Medical Students

Reasons For Willingness	Reasons Against Willingness
Altruism^23,32^	Academic commitments^24^
CV improvement and gaining future contacts^23^	Family health and safety concerns^26^
Increase self-confidence in sim situations^23^	Family/social commitments^24^
Obligation/social responsibility^23,25^	Financial implications^24^
Professional and skills development^23^	Inefficiency^26^
Reduce guilt about less fortunate^23^	Lack of confidence in skills^19^
Sense of ethics^25^	Lack of information^24^
	Needless sacrifice^26^
	Personal health and safety concerns^19^
	Work commitments^24^

**TABLE 5:** Summary of the Factors Affecting the Level of Willingness Reported by Medical Students

Factors Encouraging Willingness	Factors Discouraging Willingness
Four or less family members^25^	Increased severity (very dangerous and contagious compared to very infectious)^35,36^
Greater level of knowledge^17,19,34^	Pediatric patient care^34^
History of a severe illness to them or family member^25^	Respiratory transmission compared to contact^37^
Less than 24 y old^25^	
Medical role (compared to admin role)^23^	
More than 100 h of previous volunteering^25^	
Natural disaster (compared to an infectious disease)^23^	
PPE availability^36^	
Training before work^23^	
Travel compensation^23^	
Well run organization^23^	

When comparing willingness levels with medical students’ confidence and knowledge in the final 2 y of medical school, willingness was seen to be proportionally higher than knowledge.^17,24,34-36^ One article reported over 59% of students willing to help, but only 23.7% of students believed they have the skills to help.^23^

### Appropriateness

Thirteen articles discussed appropriateness of the roles. This included looking at medical students’ confidence and preparedness for specific roles. Five studies measured students’ skill levels; confidence to deal with emergency outbreaks ranged between 23.7 and 53.2% (Table 6).^23,26,33-35^ Three articles found that students’ willingness was high, but when compared with their self-perceived knowledge, they did not feel prepared.^33-35^ The perspective study by Starr describes “For me and my classmates, knowledge of the disease we were to face so soon was limited to the contents of that 1 lecture.”^40^

**TABLE 6:** Overview of the Students’ Confidence in Their Skills, Knowledge, and Education to Deal With Global Health Emergencies

Article ID	Context (Self-Perceived)	Percentage of Students
Gouda (2019)^23^	Sufficiently skilled to respond to an emergency outbreak	23.70
Kaiser (2009)^26^	Sufficiently skilled to respond to natural disasters	51.60
	Sufficiently skilled to respond to pandemic influenza	53.20
Mortelmans (2009)^33^	Sufficiently educated to help in H5N1 pandemic	46
Mortelmans (2013)^34^	Sufficient knowledge on disease management in disaster situations	18
Mortelmans (2015)^35^	Sufficient skills to deal with infectious outbreak	39.20

The need for disaster management training was also highlighted (*n* = 2), due to the lack of preparation of students.^18,39^ Only 3 articles reported training their students before carrying out their role, and Reyes described volunteers as being “quickly trained.”^30.31.39^ There are ethical issues of involving medical students in global health emergencies, including students’ safety, medico-legal issues, and health insurance of students.^22,29,30^ Eastwood et al. specifically highlighted the importance of students to be well informed and be able to make the decision of being involved themselves.^22^

## DISCUSSION

The review outlines the past involvement of medical students in global health emergencies. This can help to guide decision-makers in choosing appropriate roles that medical students are willing to do and are prepared to carry out in a global health emergency.

### Role

Historically, medical students have been involved in the response to global health emergencies and pandemics in a variety of ways.^16,18,21,28,37,38^ Our results identified a range of roles that students have played in the past, thus highlighting the potential roles medical students are able to play in the current COVID-19 pandemic or future global health emergencies.

Unlike members of the general public, medical students have attained several relevant clinical skills during their years of training, which can be useful in a situation where the healthcare system is under pressure. For example, in 1 study, students assisted the resuscitation team, a skill students learn during medical school.^28^ The current data suggest that the most common roles played were, in fact, clinical roles. However, nonclinical roles, such as runners to carry information, assisting in hotlines, and psychiatric services were also described (Chen E, Goodman KW, Fiore RN. Involving medical students in disaster response: ethics, education and opportunity [unpublished PDF]. 2014;1-17).^28^

A role that should not be neglected, however, is the normal role of a medical student—to learn. It is important to consider whether these alternative roles are appropriate in a specific situation. Of interest, 1 study concluded that being involved in such situations helps strengthen students’ desires to become physicians, suggesting that involvement does not only help to serve healthcare systems but the students themselves.^28^ Other studies have also found clinical volunteering to be perceived positively by medical students, helping them develop clinical skills and collaborate with other healthcare professionals.^43^

The range of roles described in the included articles highlights the versatility of medical student involvement. For example, 6 articles also described that medical students further in their medical education were more likely to provide medical assistance.^18,19,22,27,28,40^ One study found younger students were involved in more educational roles as first year students helped by raising awareness for HIV.^21^ This demonstrates that involvement in the response to global health emergencies is not limited to advanced medical students and that younger students, even though they may have less clinical knowledge, can still offer a valuable contribution when given an appropriate role. Furthermore, the variety of roles can allow certain challenges to be avoided. For instance, if a hospital is deemed to have a lack of supervisors, a commonly faced challenge even during nonemergency times, then medical students can get involved in a nondirect clinical role.^18^

Several challenges were described as students undertook these roles, including a lack of supervision, a lack of experience, and a negative impact on their mental health.^18,28,29,39^ Pro-actively addressing these challenges will not only enable any involvement medical students have in the current or any global health emergency to be efficient and effective, but will also reduce any detrimental effect on the medical students’ mental health and medical education.

Our results demonstrate that such involvement in highly stressful situations can lead to anxiety, depressive symptoms, and emotional distress.^28,29,39^ Consequently, stress can lead to a higher prevalence of university student dropout.^44^ To avoid negative mental health impacts, adequate support must be provided to medical students if they are to be involved. This also highlights the need for further research that explores the short- and long-term effects on the students involved, and more specifically, the impact on their mental health and their future careers.

Ultimately, the roles of medical students might be very context-specific. A limitation of this review is the lack of data providing specific recommendations on the most suitable role of medical students in the differing types of global health emergencies. A further limitation is the paucity of data evaluating the roles medical students have previously undertaken. It is important to ascertain how effective medical students were in the emergency response and whether they made a real impact. Therefore, further research into the effectiveness of medical student involvement would be beneficial, and perhaps comparing this to global health emergencies that have not involved medical students.

### Willingness

Eleven of the 12 articles (91.7%) discussing willingness reported a greater willingness than unwillingness among medical students to be involved in the response to pandemics and global health emergencies. Only Hwang et al. found the opposite pattern of only 36% of students willing to be involved.^25^ This article focused specifically on whether medical students would enter a hypothetical closed area with a highly infectious disease and high fatality rate. Of interest, only 9% of those not wanting to enter stated fear of safety as their reason, and in contrast, 44% were unwilling to enter due to inefficiency.^25^ This highlights the importance of organization when adding medical students to the workforce.

Obligation to help and social responsibility were the 2 most commonly stated reasons for wanting to help in such situations (Chen E, Goodman KW, Fiore RN. Involving medical students in disaster response: ethics, education and opportunity [unpublished PDF]. 2014;1-17).^23-25^ This suggests that medical students believe they “should” volunteer, rather than “want” to. In turn, this can lead to students putting pressure on themselves to help, when they may not feel ready to do so, which will potentially hinder any positive effect they may have on the disaster situation, and could be dangerous for patients. Making students aware of both their mental and physical capabilities is essential to ensure that they only volunteer if they are prepared.

Certain factors encouraged student involvement, including a greater level of knowledge and being trained and receiving travel compensation. The most common discouraging factor was the severity of the event or outbreak.^17,19,23,34,35^ It is important to consider these factors, specifically by emphasizing encouraging factors, such as travel compensation and training before work. This, along with minimizing any discouraging factors where possible, will ensure the maximum number of medical students will be prepared to volunteer and, thus, increase the size of the workforce.

It is vital to support medical students to gain the extra help that is needed, as medical students would be going beyond their role of simply learning and being a student in an already stressful situation. Considering ways to decrease stress can help reduce negative mental health effects both during and after the event. Factors such as age and previous volunteering experience can be used to help target a specific cohort of medical students to volunteer with specific roles. For instance, initially approaching medical students who are already experienced with volunteering, before approaching the remainder of the medical student cohort if required, may be a more beneficial way of organizing a volunteering scheme.

Several studies found students reported that their willingness was higher than their knowledge and readiness.^17,23,33-35^ This illustrates the need to better prepare medical students, who can indeed be a great addition to the workforce in disastrous situations. Knowledge about a specific disease is vital to students undertaking both clinical and nonclinical roles, such as raising awareness.^45^ This preparation could be spread throughout their medical school education, or delivered immediately before a specific role.^46,47^ The latter, however, would require further resources acutely during a disease outbreak, which may not be available.

The studies discussing willingness were limited by their methodology as they only measured medical students’ responses and did not compare with the general population. Furthermore, most studies were hypothetical situations and, therefore, students may answer differently if the real situation arose. Furthermore, there was no standardized survey of measuring willingness between all studies and this, along with the studies not comparing to a general cohort, limited the ability to carry out a meta-analysis.

### Appropriateness

Three articles reported that, although medical students may have been willing to help, they may not have necessarily felt prepared or felt that they knew enough.^33-35^ The ethical considerations of the roles of medical students must be thoroughly explored before students are invited to help, especially where patient care may be compromised.

During times of nonglobal health emergencies, the primary role of medical students is education and to learn to be a doctor, which can take between 4 and 7 y. The need to help the workforce in times of crisis must be balanced with the educational and wellbeing needs of the medical student to complete their training. Whereas “learning on the job” can be an invaluable experience, the safety of both medical students and their patients must be considered. However, there was considerably less data on this aspect in the evidence base.

## STRENGTHS AND LIMITATIONS OF ARTICLE

This rapid systematic review provides an overview of the previous involvement of medical students in global health outbreaks, which, to the authors’ knowledge, no other systematic review has previously discussed. The inclusion criteria were kept broad, which allows the results to be generalizable for other future global-health emergencies, as well as the current COVID-19 pandemic. The predefined aims and objectives were answered and previous roles and their appropriateness were summarized. Although completing a rapid review may help inform the current COVID-19 pandemic, this study design also has limitations, especially due to time constraints. First, although each included article was discussed and data were extracted by 2 authors, no full formal critical appraisal or risk of bias tool was used. Second, due to the data not being sufficiently homogenous, a meta-analysis was not possible. Third, gray literature was included, which may yield lower quality evidence; however, on balance, it provided an invaluable insight into previous roles that had taken. The fact that medical students are the authors of this review is both a strength and a limitation. The authors are themselves experiencing the possibility of being involved in the current COVID-19 pandemic and, therefore, have insights into the difficulties and lack of data about this process.

### Implication for Policy and Practice

Much has been written on previous roles students have taken in previous situations, and this can be used to inform future policy regarding COVID-19 and future global health emergencies. When designing the role itself, the willingness and preparedness of the medical students should be strongly taken into account. This will ensure specific roles are safe and within appropriate student competencies. Medical students should be given clinical roles within reason, and educational and social media roles may be given for the less clinically confident. This highlights the importance of co-production and including students themselves when planning these roles.

**FIGURE 2:**
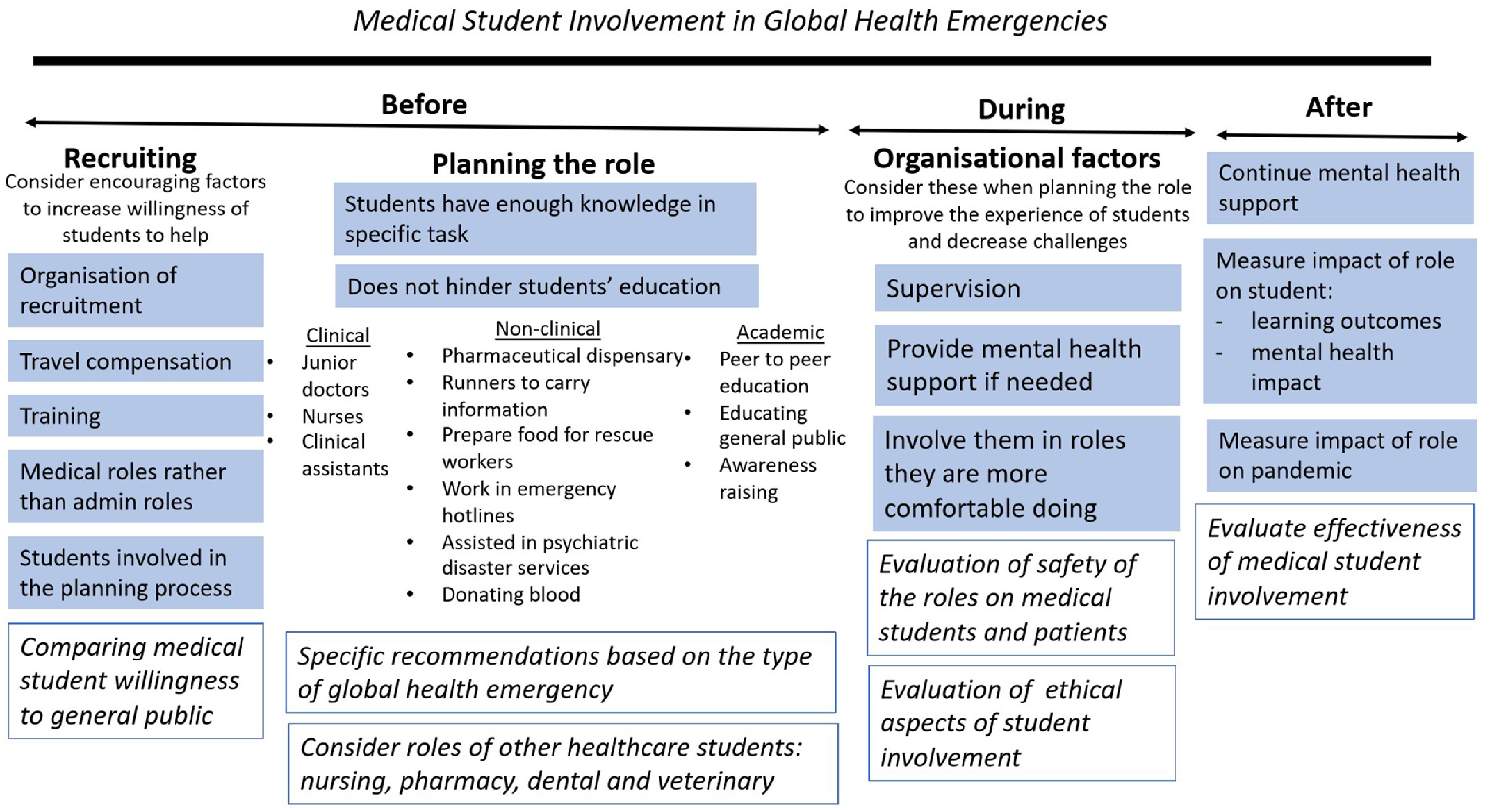
Medical Student Involvement in Global Health Emergencies.

The authors of this review experienced firsthand the difficulty in defining roles for medical students in the current COVID-19 pandemic, within their own universities and hospital trusts. This highlights the lack of robust policy and knowledge surrounding this topic, and the consequent unnecessary delay in the use of a skillful resource. We thoroughly encourage governments to have predefined policies for medical schools if such events arise again. An infographic was made to summarize the findings of this review (Figure 2).

## FUTURE RESEARCH

Future research to both describe and evaluate the effectiveness of medical students’ roles during the current pandemic should be carried out to help guide future pandemics. This research should also encompass the safety of these roles and the short- and long-term effects on the medical students themselves. Furthermore, there is a dearth of literature on the ethical aspects of medical student involvement and duty during such situations. This has been discussed when looking at residents/junior doctors participating in such situations, but not for the medical student cohort.^48^ Finally, this area of research can be expanded to provide information on the role of other healthcare students in global health emergencies, namely nursing, pharmacy, dental, and veterinary students.

## CONCLUSIONS

Medical students are a willing and resourceful potential addition to the healthcare workforce during global health emergencies. Their involvement is vast and many roles have been identified; however, adequate and proactive support must be provided to help them overcome any challenges they may face. Choosing the perfect role is very subjective to each emergency. Therefore, it is vital to consider available resources, students’ opinions, and the nature of the emergency itself when planning roles. Future research should be targeted at filling important gaps in the literature discussed above, including evaluating the effectiveness of different roles undertaken by medical students in global health emergencies and the ethical issues regarding the appropriateness of the medical students’ involvement.

## APPENDIX 1

PUBMED: ((medical student[Title/Abstract] OR student doctor[Title/Abstract] OR medical student*[Title/Abstract])) AND (Pandemic[Title/Abstract] OR Disease outbreak[Title/Abstract] OR Disaster medicine[Title/Abstract] OR Epidemic[Title/Abstract] OR Acute Respiratory Infection[Title/Abstract] OR Severe Acute Respiratory Syndrome[Title/Abstract] OR Global health emergency[Title/Abstract] OR Public health emergency[Title/Abstract])

OVID: ((medical student or student doctor or medical student*) and (Pandemic or Disease outbreak or Disaster medicine or Epidemic or Acute Respiratory Infection or Severe Acute Respiratory Syndrome or Global health emergency or Public health emergency)).ab,ti.
